# Prurigo Pigmentosa (Keto Rash) Secondary to an Eating Disorder: A Case Report and Proposed Diagnostic Criteria

**DOI:** 10.7759/cureus.90225

**Published:** 2025-08-16

**Authors:** Mohammed Shanshal, Emma Jackson

**Affiliations:** 1 Dermatology, Imperial College Healthcare NHS Trust, London, GBR

**Keywords:** dietary dermatoses, ketogenic diet, keto rash, prurigo pigmentosa, reticulated hyperpigmentation

## Abstract

Prurigo pigmentosa is a rare inflammatory dermatosis increasingly reported in association with ketogenic diets, fasting, diabetes, and eating disorders. We describe a 21-year-old female admitted after an intentional overdose of nonsteroidal anti-inflammatory drugs (NSAIDs) and aspirin, who presented with a pruritic, symmetric erythematous papular eruption involving the trunk, neck, upper arms, and back, which evolved into a reticulated hyperpigmented pattern. Urinalysis confirmed ketonuria. Histology demonstrated chronic lymphohistiocytic perivascular inflammation, melanophages, and pigment incontinence, consistent with the evolving subacute prurigo pigmentosa. This case highlights a frequently misdiagnosed eruption that can mimic common dermatoses. To address this diagnostic challenge, we propose a set of practical diagnostic criteria based on clinical features, disease course, and the context of ketosis. Early recognition, aided by these criteria, is essential for effective management through dietary modifications, thereby reducing unnecessary investigations and facilitating prompt resolution.

## Introduction

Prurigo pigmentosa is a rare inflammatory dermatosis first described by Nagashima in Japan in 1971 [1]. It predominantly affects young females, with a predilection for Asian populations, though increasing numbers of cases have been reported worldwide [2]. The exact pathogenesis remains unclear, but the condition is strongly associated with states of ketosis, including dietary restriction, fasting, diabetes mellitus, and eating disorders. With the global rise in the adoption of the ketogenic diet, prurigo pigmentosa has become increasingly relevant in clinical practice [3].

Clinically, the eruption typically begins as pruritic, symmetric erythematous papules and plaques on the trunk, neck, and proximal limbs, evolving into a reticulated inflammatory pattern and ultimately resolving with residual hyperpigmentation. Histopathology reveals stage-dependent features, including spongiosis and a superficial perivascular infiltrate in early lesions and a lichenoid infiltrate with pigment incontinence and melanophages in later stages [4].

Prurigo pigmentosa is often misdiagnosed as more common dermatoses, such as viral exanthems, pityriasis rosea, or confluent reticulated papillomatosis, contributing to delays in recognition and appropriate management. Awareness of the clinical presentation, epidemiology, histopathological features, and association with ketosis is essential, as simple dietary modification can result in complete resolution without pharmacologic intervention [5].

This report describes a classic case of prurigo pigmentosa triggered by an eating disorder to illustrate its clinical evolution and underscores the diagnostic utility of our proposed clinical criteria in preventing misdiagnosis.

## Case presentation

**Figure 1:**
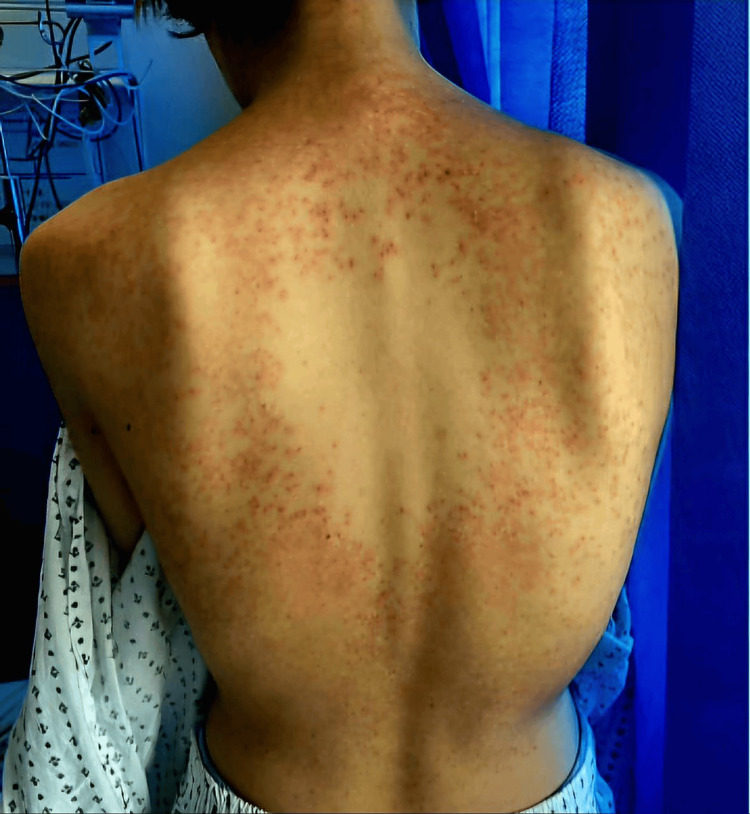
Symmetrical erythematous papules and plaques distributed over the back in a reticulated pattern, characteristic of prurigo pigmentosa (keto rash)

**Figure 2:**
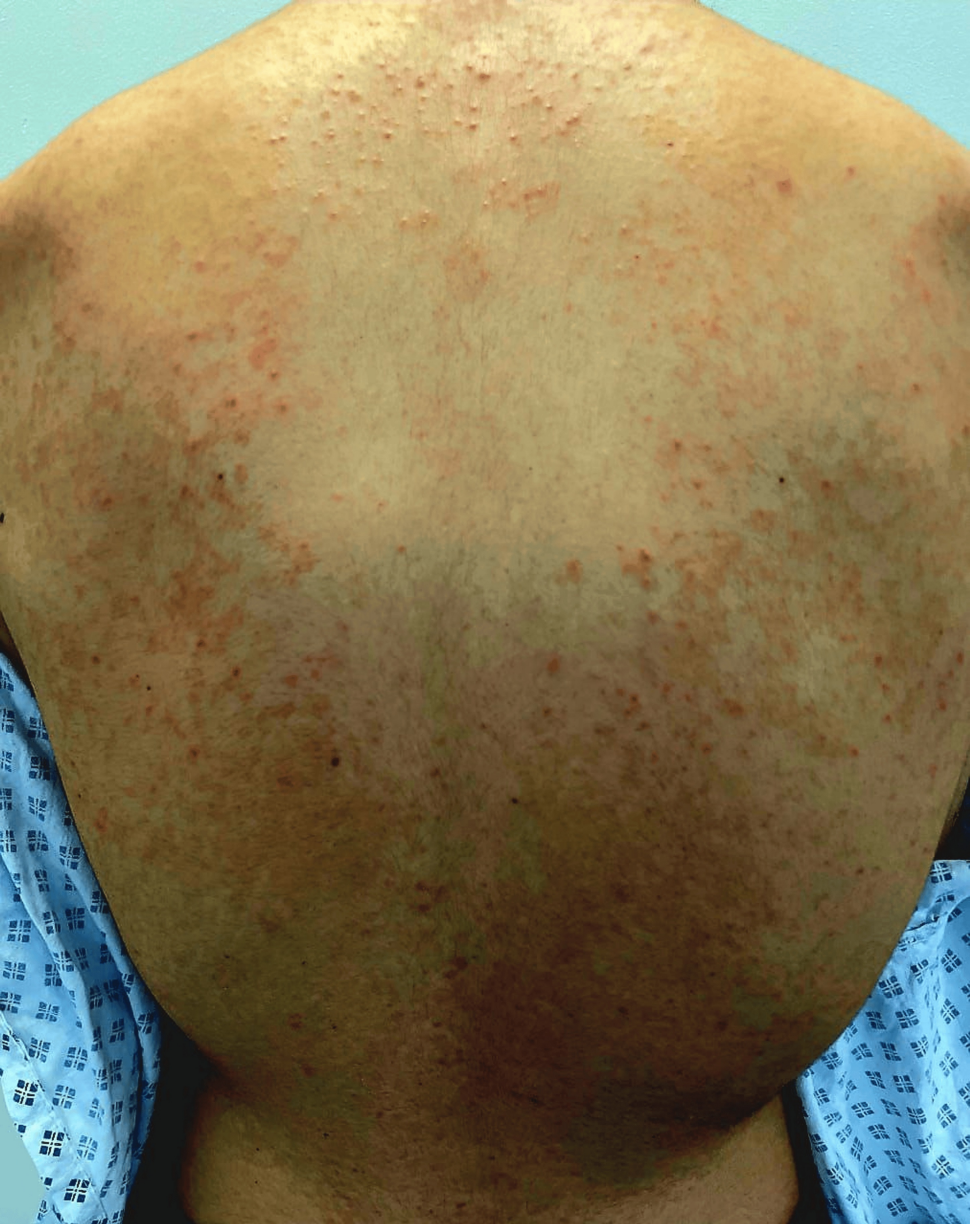
Close-up view of erythematous papules and early pigmentation on the upper back

**Figure 3:**
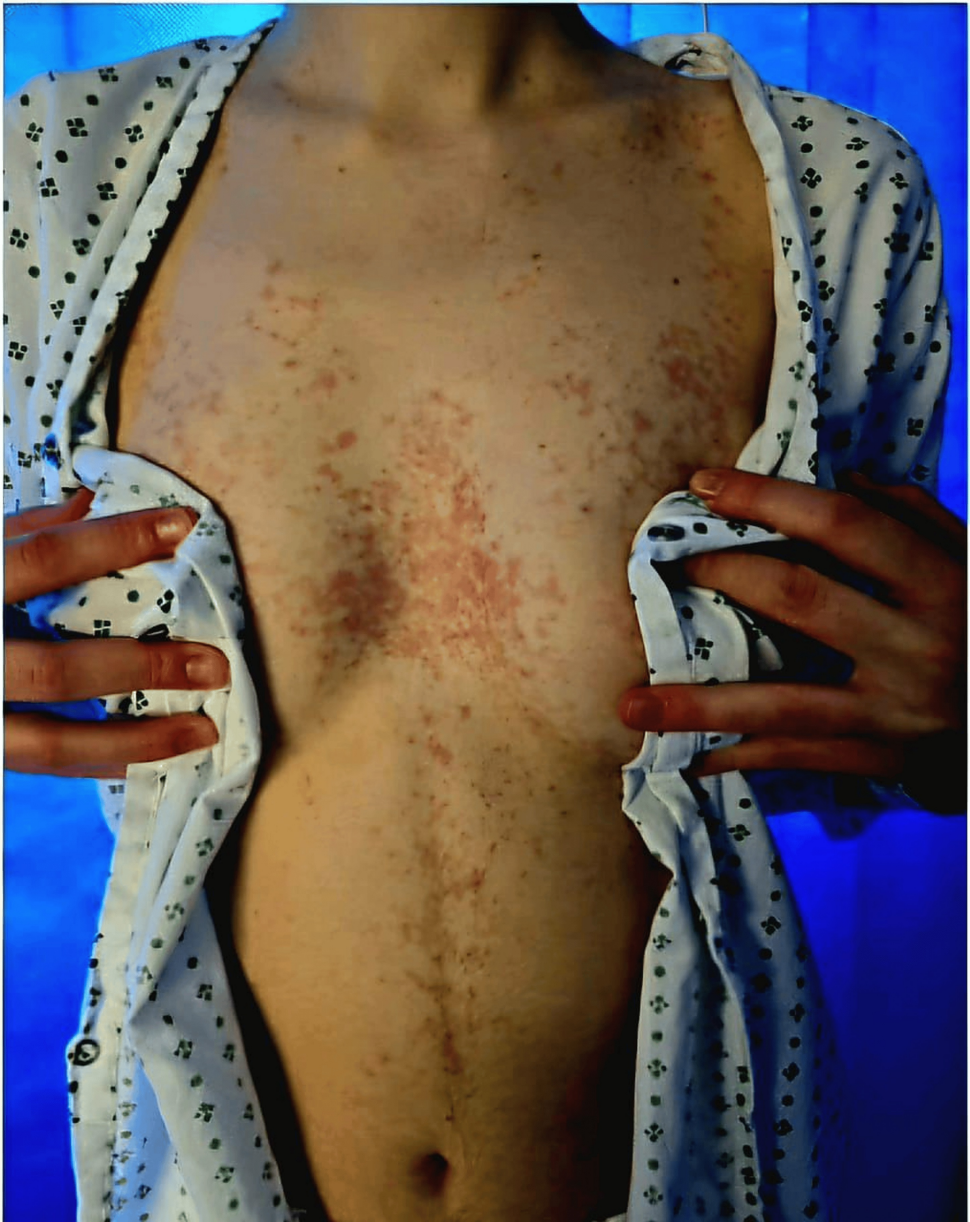
Anterior view showing symmetric erythematous papules and reticulated hyperpigmentation over the chest and upper abdomen in a patient with prurigo pigmentosa.

A 21-year-old female was admitted following an intentional overdose of nonsteroidal anti-inflammatory drugs (NSAIDs) and aspirin. Her medical history included obsessive-compulsive disorder and an eating disorder. Dermatology was consulted for an itchy rash that had developed over several weeks prior to admission. Examination revealed a symmetric eruption of erythematous papules affecting the torso, neck, upper arms, and back (Figures 1-3).

The eruption was first thought to represent a viral exanthem but evolved into a reticulated pattern with hyperpigmentation over subsequent days. Urinalysis confirmed the presence of ketone bodies (ketonuria: 4+ on dipstick).

A punch biopsy of an active lesion demonstrated mild chronic lymphohistiocytic perivascular inflammation, melanophages, and pigment incontinence, consistent with subacute evolving prurigo pigmentosa. Following dietary counseling and the reintroduction of a balanced diet, the pruritus subsided within one week, and the erythematous papules began to fade, leaving post-inflammatory hyperpigmentation.

## Discussion

Prurigo pigmentosa (PP) is a distinctive inflammatory dermatosis whose recognition is increasingly critical in an era of popular diet-induced ketosis. The case of the 21-year-old female presented here, with a background of an eating disorder leading to ketonuria, classically illustrates the clinical progression and diagnostic challenges of this condition. The evolution from an eruption of pruritic erythematous papules, initially mistaken for a viral exanthem, to a characteristic reticulated hyperpigmented pattern is pathognomonic for PP and underscores the importance of considering this diagnosis in the appropriate clinical context.

While its exact pathogenesis is not fully understood, the condition is strongly linked to ketotic states. Leading theories suggest that ketone bodies may have a direct pro-inflammatory effect on the skin or that ketosis-induced changes in the gut microbiota could trigger a systemic inflammatory response manifesting cutaneously [6].

Diagnosing PP involves recognizing its classic clinical evolution through distinct stages: an initial phase characterized by erythematous papules or plaques, a second phase of reticulated inflammatory pigmentation, and a late phase of resolution leaving residual hyperpigmentation. Early lesions may mimic viral exanthems, pityriasis rosea, or confluent reticulated papillomatosis, leading to diagnostic delay [7]. The diagnosis is primarily clinical, supported by a history of a ketotic state (confirmed by urinalysis), and can be aided by histopathology, which varies with the lesion's age, with early lesions demonstrating spongiosis and superficial perivascular infiltrate, while later lesions exhibit lichenoid inflammation, pigment incontinence, and melanophages [8,9]. To aid clinicians in navigating this diagnostic challenge, we propose a set of practical diagnostic criteria based on our case and a review of the literature (Table 1).

**Table 1:** Proposed diagnostic criteria for prurigo pigmentosa The diagnosis of prurigo pigmentosa is supported when most criteria are met, typically at least four of five, particularly in the context of ketosis.

Criterion	Description
1. Characteristic clinical features	Pruritic, symmetric erythematous papules or plaques predominantly on the trunk, neck, and proximal limbs, evolving into a reticulated pattern and resolving with hyperpigmentation
2. Typical course	Acute or subacute onset with progression over days to weeks and resolution leaving post-inflammatory pigmentation
3. Triggering context	Clinical history consistent with a ketosis-related state (e.g., dietary restriction, diabetes mellitus, eating disorder) and/or confirmed ketonuria
4. Supportive histopathology	Stage-dependent histopathologic features, if biopsy performed (e.g., spongiosis, superficial perivascular infiltrate, pigment incontinence)
5. Exclusion of mimickers	Exclusion of pityriasis rosea, viral exanthems, confluent reticulated papillomatosis, and other reticulated dermatoses

Our patient fulfilled all five proposed criteria: (1) She presented with the characteristic pruritic, symmetric papules evolving into a reticulated pattern; (2) The course was subacute; (3) A clear triggering context of an eating disorder and confirmed ketonuria was present; (4) Histopathology was supportive; and (5) Common mimickers were considered and excluded as the clinical picture evolved.

The cornerstone of management is the reversal of the underlying ketosis, most effectively achieved by increasing carbohydrate intake. This dietary modification typically leads to a rapid cessation of pruritus and prevents new lesions. For symptomatic relief, particularly for severe itching, tetracycline-class antibiotics like doxycycline or minocycline are considered first-line pharmacologic agents due to their potent anti-inflammatory properties. While other treatments like dapsone exist for refractory cases, the primary goal remains the identification and correction of the metabolic trigger [10,11].

## Conclusions

Prurigo pigmentosa is a distinctive, ketosis-associated inflammatory dermatosis that, while uncommon, is of growing importance for clinicians to recognize in various specialties, including dermatology, internal medicine, and emergency medicine. This case highlights the classic presentation in a young female with an eating disorder, a context where ketosis is a frequent metabolic consequence. The critical takeaway is that a high index of suspicion in patients presenting with a pruritic, symmetric, and reticulated eruption on the trunk should prompt an inquiry into recent dietary habits and a simple urinalysis for ketones.

Early and accurate diagnosis, facilitated by the clinical criteria we have outlined, can prevent a cascade of unnecessary investigations, avoid the prescription of ineffective treatments like topical steroids, and lead to prompt resolution. Management is often straightforward, hinging on the simple, yet highly effective, strategy of dietary carbohydrate reintroduction. As ketogenic and other restrictive diets continue to gain popularity for various health and lifestyle reasons, increased clinical awareness of 'keto rash' is essential to ensure timely and appropriate patient care.
